# Sequence variants identification at the *KCNQ1OT1*:TSS differentially Methylated region in isolated omphalocele cases

**DOI:** 10.1186/s12881-017-0470-z

**Published:** 2017-10-18

**Authors:** Maria Francesca Bedeschi, Mariarosaria Calvello, Leda Paganini, Lidia Pezzani, Marco Baccarin, Laura Fontana, Silvia M. Sirchia, Silvana Guerneri, Lorena Canazza, Ernesto Leva, Lorenzo Colombo, Faustina Lalatta, Fabio Mosca, Silvia Tabano, Monica Miozzo

**Affiliations:** 1 Clinical Genetics Unit, Fondazione IRCCS Ca’ Granda Ospedale Maggiore Policlinico, Milan, Italy; 2 Division of Pathology, Fondazione IRCCS Ca’ Granda Ospedale Maggiore Policlinico; Department of Pathophysiology & Transplantation, Università degli Studi di Milano, Milan, Italy; 3 Medical Genetics Laboratory, Fondazione IRCCS Ca’ Granda Ospedale Maggiore Policlinico, Milan, Italy; 4 Department of Health Science, Università degli Studi di Milano, Milan, Italy; 5 Department of Pediatric Surgery, Fondazione IRCCS Ca’ Granda Ospedale Maggiore Policlinico, Milan, Italy; 6 Neonatal Intensive Care Unit, Department of Clinical Science and Community Health, Università degli Studi di Milano and Fondazione IRCCS Ca’ Granda Ospedale Maggiore Policlinico, Milan, Italy

**Keywords:** Abdominal wall defects, Beckwith-Wiedemann syndrome, *CDKN1C*, Genomic imprinting, *KCNQ1OT1*:TSS-DMR, Omphalocele

## Abstract

**Background:**

Omphalocele is a congenital midline ventral body wall defect that can exist as isolated malformation or as part of a syndrome. It can be considered one of the major and most frequent clinical manifestation of Beckwith-Wiedemann Syndrome (BWS) in case of loss of methylation at *KCNQ1OT1*: Transcription Star Site-Differentially Methylated Region (TSS**-**DMR) or in presence of *CDKN1C* mutations. The isolated form of the omphalocele accounts approximately for about the 14% of the total cases and its molecular etiology has never been fully elucidated.

**Methods:**

Given the tight relationship with BWS, we hypothesized that the isolated form of the omphalocele could belong to the heterogeneous spectrum of the BWS associated features, representing an endophenotype with a clear genetic connection. We therefore investigated genetic and epigenetic changes affecting BWS imprinted locus at 11p15.5 imprinted region, focusing in particular on the *KCNQ1OT1*:TSS DMR.

**Results:**

We studied 21 cases of isolated omphalocele detected during pregnancy or at birth and identified the following rare maternally inherited variants: i) the non-coding variant G > A at nucleotide 687 (NR_002728.3) at *KCNQ1OT1*:TSS-DMR, which alters the methylation pattern of the imprinted allele, in one patient; ii) the deletion c.624-629delGGCCCC at exon 1 of *CDKN1C*, with unknown clinical significance, in two unrelated cases.

**Conclusions:**

Taken together, these findings suggest that *KCNQ1OT1*:TSS-DMR could be a susceptibility locus for the isolated omphalocele.

**Electronic supplementary material:**

The online version of this article (10.1186/s12881-017-0470-z) contains supplementary material, which is available to authorized users.

## Background

Omphalocele is a congenital midline ventral body wall defect characterized by the extrusion of abdominal viscera through the base of the umbilical cord. This malformation is related to the failure of the midgut loop to return to the peritoneal cavity after its herniation into the umbilical cord [1]. The incidence of omphalocele diagnosed during pregnancy is approximately 1/5000; however, this falls to 0.8/10,000 live births because of pregnancy termination [2].

The definition of abdominal wall defects can be difficult, since a ruptured omphalocele may be misdiagnosed as gastroschisis and an umbilical cord hernia can be confused with a small omphalocele. Gastroschisis is caused by vascular defects, does not involve the insertion site of the umbilical cord, and is generally localized to the right of the umbilicus [3]. Conversely, omphalocele results from an enlargement of the umbilical ring due to the migration failure of cephalic and/or lateral folds and abdominal bands during early embryogenesis [4, 5].

**Fig. 1:**
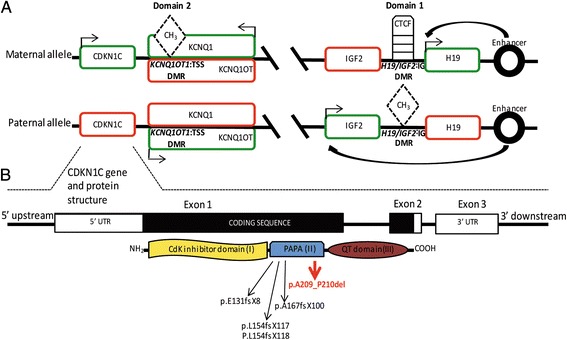
Epigenetic and genetic regulation of the 11p15.5 imprinted locus. As shown in (**a)**, the 11p15.5 imprinted locus presents with 2 domains (Domain 1 and Domain 2) differentially regulated by *H19/IGF2-*IG and *KCNQ1OT1*:TSS DMRs, respectively. Transcribed genes are depicted in green, silenced genes in red. On the maternal allele, the effect of the enhancer promotes the expression of *H19,* the most proximal gene, but not of *IGF2*. This is the consequence of the CTCF binding to *H19/IGF2-*IG DMR located between the two genes. On the paternal allele, methylation of this DMR prevents the binding of CTCF, allowing the expression of *IGF2* and the silencing of *H19*. At *KCNQ1OT1*:TSS DMR the opposite methylation pattern is observed. This DMR is methylated on the maternal allele and not on the paternal one. Thus, maternal repression of *KCNQ10T* allows the expression of *CDKN1C* and *KCNQ1,* while the expression of the *KCNQ10T* on the paternal allele inhibits the transcription of the flanking genes *CDKN1C* and *KCNQ1. CDKN1C* gene and protein structure are depicted in (**b)**. White striped boxes identify the 5′- and 3′-UTRs and the black portion represents the coding sequence of the gene. *CDKN1C* encodes for P57 protein (alias KIP2), composed of the three functional domains depicted with different colors. Black arrows indicate previously identified truncating mutations inside the PAPA domain; red arrow points out the susceptibility variant p.A209_P210del identified in this paper

Omphalocele may be syndromic or isolated. Of syndromic cases, 54–57% are caused by aneuploidy, with a prevalence of trisomy 18 [6]. The remaining cases are associated with a number of genetic conditions including Beckwith-Wiedemann syndrome (BWS; MIM 130650), pentalogy of Cantrell (OMIM 313850), and OEIS (Omphalocele, Exstrophy, Imperforate anus, Spinal defects; OMIM 258040) [7–9]. Of these, BWS is the most common syndrome associated with omphalocele [10]. Indeed, isolated omphalocele can be the first manifestation of a mild clinical form of BWS, in a similar way to isolated hemihyperplasia, which can be predictive of BWS [11, 12]. BWS is genetically heterogeneous and may be caused by genomic, epigenetic, or genetic alterations at the 11p15.5 chromosomal locus. This region harbors two imprinted domains, *IGF2/H19* and *KCNQ1/CDKN1C*, regulated by *H19/IGF2:*IG-DMR (InterGenic - Differentially Methylated Region) and *KCNQ1OT1*:TSS-DMR (Transcription Start Site – Differentially Methylated Region), respectively. In healthy individuals, *H19/IGF2*:IG-DMR is methylated on the paternally derived allele and unmethylated on the maternally inherited allele, resulting in the preferential expression of the paternal *IGF2* and the maternal *H19*. At *KCNQ1OT1*:TSS-DMR, the opposite methylation pattern is observed [11, 12]. The *KCNQ1OT1*:TSS-DMR regulates the expression of *CDKN1C* and *KCNQ1*, as well as other genes, including *PHLDA* and *SLC22A18*. *KCNQ1OT1*:TSS-DMR is located within intron 10 of *KCNQ1* and encompasses the promoter of the non-coding RNA *KCNQ1OT1*, which is orientated in the antisense direction relative to *KCNQ1*. Fig. 1a shows a schematic representation of the imprinted locus. Physiological expression of *KCNQ1OT1* from the unmethylated paternal allele results in the bi-directional silencing of its flanking imprinted genes [13]. Among them, *CDKN1C* encodes P57 (alias *KIP2*), a tight-binding inhibitor of several G1 cyclin/Cdk complexes, acting as a negative regulator of cell proliferation [14]. The *CDKN1C* gene contains three exons and 2 GC-rich introns; however, only exons 1 and 2 encode the functional P57 protein. P57 is composed of 316 amino acids and consists of three structurally distinct domains: (i) the N-terminal domain (aa 1–110), which has significant similarity to the CdK-inhibitors, p21^Cip1^ and p27^Kip1^, and is necessary for CdK inhibition; (ii) the central highly polymorphic hexanucleotide repeat domain, encoding a series of proline-alanine repeats (PAPA-repeats, aa 156–213) involved in Mitogen-Activated Protein Kinase (MAPK) phosphorylation [15]; and (iii) the highly conserved C-terminal region (QT domain) that exhibits homology with p27^Kip1^ [16].

Decreased *CDKN1C* activity due to genetic variants, loss of *KCNQ1OT1*:TSS-DMR maternal methylation, or changes in gene copy number is associated with BWS. Forty to 50 % of BWS cases present with loss of *KCNQ1OT1*:TSS-DMR maternal methylation [17]. Abdominal wall defects are very common in cases with hypomethylation: 42.1% of patients have omphalocele, 20% present with umbilical hernia, and approximately 12% with diastasis recti [18].

Concerning genetic variants, causative *CDKN1C* point mutations are maternally inherited and cause protein loss-of-function. Point mutations of *CDKN1C* are present in up to 5% of sporadic and 50% of familial cases of BWS [19]. Most frequently, *CDKN1C* mutations are truncating or frameshift and are distributed along the entire coding region; conversely, missense mutations are mainly localized in the CDK-like domain [14]. Almost 90% of BWS patients with *CDKN1C* mutations have abdominal wall defects [18]; in particular, omphalocele occurs in 71.6% of these cases [18]. Finally, structural chromosome alterations such as deletions/duplications (1–2% of BWS cases) and uniparental disomy (UPD) at 11p15.5 involving *CDKN1C* (10–20% of BWS cases) [15] cause umbilical hernias in about half of patients, whereas in others no abdominal wall defects are described [18]. Thus, omphalocele can be considered one of the major and most frequent clinical manifestations of BWS in cases of *KCNQ1OT1*:TSS-DMR loss of methylation or *CDKN1C* mutations [20, 21].

A well-defined (epi)genotype–phenotype correlation exists for omphalocele in BWS: it is caused by *KCNQ1OT1*:TSS-DMR loss of methylation in 86% of the cases, by *CDKN1C* mutation in 7%, and by UPD or *H19/IGF2*:IG-DMR gain of methylation in <10% of patients [18].

Approximatively 14% of all cases of omphalocele can be classified as isolated [2]. Despite several studies investigating the genetic causes [22–25] or environmental risk factors [24] involved in this malformation, the etiology of isolated omphalocele has not been fully elucidated. Kanagawa et al. [23] described an extensive family with vertical transmission of isolated omphalocele, with nine subjects over three generations affected, suggesting an autosomal dominant pattern of inheritance. This finding was in agreement with five other previously reported pedigrees demonstrating vertical transmission of isolated omphalocele [25]. The same authors also described five families with an inheritance consistent with an autosomal recessive pattern [25]. Despite these observations, inherited alterations have never been identified in these families. More recently, a genomic duplication was found in a large pedigree with autosomal dominant transmission of isolated omphalocele [22]. Genetic analysis revealed that all affected individuals were positive for a duplication at chromosome 1p31.3, suggesting the involvement of one or more genes in the duplicated region, including *FOXD3*, *ALG6*, *ITGB3BP*, *KIAA1799*, *DLEU2L*, *PGM1*, and the proximal portion of *ROR1* [22].

## Methods

The early genetic characterization of newborns with omphalocele initially defined as isolated, together with a careful follow-up, can help pre- and/or post-natal clinical management and possibly enable definition of recurrence risk.

We studied 21 cases with isolated omphalocele diagnosed during pregnancy or at birth and we investigated molecular changes associated with BWS to determine whether isolated omphalocele could belong to the heterogeneous spectrum of BWS associated features. We did not consider other conditions (Pentalogy of Cantrell and OEIS) since the affected individuals usually present with complex and more severe phenotypes. Finally, we investigated whether genomic alterations at 1p31 or at other chromosome regions could be associated with this clinical manifestation.

### Participants

The study included 21 patients (10 females and 11 males) with isolated omphalocele confirmed at birth, examined from 2013 to 2015 at the Department of Pediatric Surgery of Fondazione IRCCS Ca′ Granda Ospedale Maggiore Policlinico, Milan (Italy). Patients came to the attention of the pediatric clinicians of our hospital for diagnostic purposes in the period specified above. They (or their parents, in case of patients were underage) gave their consent to the possible future use of their genetic material for research purposes.

The clinical features of the population are described in Table 1. In details, seven cases were diagnosed at birth and 14 during prenatal ultrasound screening (US). Gestational age at diagnosis, in these latter cases, ranged from 12 to 26 Weeks of gestation (Wog).

**Table 1:** Clinical features of the population involved in the study

Case Nr	Conception	Omphalocele sonographic diagnosis (Wog)	Other sonographic features (Wog)	Delivery Type (Wog)	Omphalocele features	APGAR SCORE 1′/5’	Other main clinical findings	Birth Weight (Centile)	Birth Lenght (Centile)	Birth OFC (Centile)	Follow-up		
											Age (Months)	Height/lenght (Centile)	Weight (Centile)
1	S	20	no	CS (37^+5^)	minor	9/10	no	75°	75°	75°	12	75°	75°-90°
2	ICSI, egg donation	20	no	V (38)	medium	9/10	slight lingual protrusion; folded helix	50°-75°	75°	nd	60	50°-75°	25°-50°
3	S	ND	no	CS (40)	minor	9/10	ileal atresia	75°-90°	95°	75°	18	75°	75°
4	S	20	no	CS (39)	medium	9/10	hepatic cyst	3°-5°	<<3°	10°	60	25°-50°	3°
5	S	ND	no	V (37)	minor	8/9	no	10°	10°-25°	10°	66	10°-25°	25°
6	ICSI	26	no	CS (34)	minor	6/7	neonatal hypoglycemia (20 mg/dl)	25°-50°	25°	10°	60	10°-25°	25°
7	S	20	no	CS (38)	minor	9/10	no	10°	10°-25°	na	54	50°	25°-50°
8	S	12	no	V (40)	minor	9/10	no	50°	50°-75°	na	29	25°-50°	25°-50°
9	S	ND	no	V (39)	minor	9/10	no	10°-25°	10°-25°	10°-25°	84	50°	25°-50°
10	S	ND	no	CS (39)	medium	9/10	right bowel atresia	50°	10°	50°-75°	5	50°	10°
11	IVF (twin pregnancy)	ND	funicular cyst (16th)	V (37)	minor	9/10	uracal anomalies	5°-10°	na	na	6	50°	25°-50°
12	S	22	no	CS (38)	minor	9/10	Meckel’s diverticulum	75°	na	na	22	10°	25°
13	S	13	no	CS (38)	giant	5/8	interatrial defect	50°	na	na	22	25°	<3°
14	S	12	no	CS (38)	giant	8/9	no	50°	na	na	3	25°	25°
15	S	20	no	V (36)	minor	9/10	slight left foot nails hypoplasia	5°-10°	na	na	12	50°	50°
16	S	19	no	V (39)	minor	9/10	no	50°	na	na	8	50°	75°
17	S	ND	no	V (39)	minor	10/10	no	90°	na	na	8	10°-25°	10°-25°
18	S	12	no	CS (36)	medium	4/8	no	<5°	na	na	6	10°-25°	25°
19	S	16	no	CS (37)	minor	8/10	no	3–10°	10°	10°	3	10°-50°	10°-50°
20	S	20	multiple cysts of umbilical cord	CS (33)	minor	8/9	multiple cysts of umbilical cord	10–25°	na	na	15	50°	<3°
21	S	ND	no	CS (39)	minor	9/10	no	10°	10–25°	25°	6	50°-75°	50°-75°

For each of the 21 cases involved in the study, table summarizes the type of conception, the time of the US omphalocele diagnosis, the presence of other US features, time and type of the delivery, classification of the omphalocele, the APGAR score at birth, the presence of other clinical findings, the weight, the length and the occipital frontal circumference at birth expressed as percentile, and the follow up data

*CS* Caesarian Section, *ICSI* Intra-Cytoplasmic Sperm Injection, *IVF* In Vitro Fertilization, *S* Spontaneous, *V* Vaginal, *Wog* weeks of gestation, *OFC* Occipital Frontal Circumference

*ND* Not Detected, *NP* Not Performed

The omphaloceles were classified as minor (diameter < 5 cm), medium (diameter = 5 cm) or “giant” (diameter > 5 \cm), according to literature [26]. Of the 15 cases with minor omphalocele, seven were diagnosed at birth. Only two fetuses (C11 and C20) were detected with both omphalocele and umbilical cord abnormalities (funicular cyst and multiple cysts) (Table 1).

Conception was spontaneous in 19 cases. Two cases, C2 and C6, were conceived by ICSI (intracytoplasmic sperm injection), with egg donation in C2.

Twenty of 21 pregnancies were singleton. One, C20, was a monochorionic diamniotic twin gestation in which only one twin had omphalocele and the other twin died at 32 weeks of gestation (Intrauterine Fetal Death) without exhibiting omphalocele or other evident malformations; the cause of death was unknown.

Consanguinity was reported in case 16 (the parents were first cousins).

Since omphalocele can be isolated at birth but additional signs may develop during the postnatal period, we followed up all babies for 3–84 months (Table 1). An accurate follow-up is very important and helps the correct diagnosis. Three newborns presented at birth with additional signs, but these spontaneously resolved or were considered aspecific features at the follow-up. In details, C13 had a small interatrial defect that closed spontaneously during the first year of life. Given the high prevalence of non-syndromic atrial defects (4.1:1000 live births), we considered this as a chance association, rather than an indication of a syndromic presentation [27]. C2 presented with slight lingual protrusion without true macroglossia, and helix indentation. Follow-up at 48 months did not reveal any additional signs, and the lingual protrusion spontaneously resolved. C6 had neonatal hypoglycemia, mild neonatal hypotonia, and a mild delay in the acquisition of motor skills (first steps at 19 months); at 60 months, neurological development was normal. Importantly, none of patients showed additional clinical signs associated with BWS. Maternal and paternal age at the conception ranged from 26 to 41 and from 28 to 48 years old, respectively. Other known environmental risk factors [10] were also considered and reported in the Additional file 1.

In study design/implementation STROBE guidelines were followed.

### DNA extraction

Genomic DNA was extracted from amniotic fluid (case 15) or peripheral blood lymphocytes (PBL; all other cases and controls), using the QIAamp DNA Mini Kit (Qiagen, Redwood City, CA, USA) according to the manufacturer’s protocol. We also analyzed DNA from PBL of 50 healthy controls, to investigate Variants of Unknown Significance (VUS) in *CDKN1C*, and from relatives of cases 16, 19, and 20, to investigate the inheritance pattern of genetic variants found in the probands. DNA from the deceased twin of case 20 was extracted from formalin-fixed paraffin-embedded (FFPE) material obtained at autopsy, using a FFPE Nucleic Acid Extraction and Purification Kit (Covaris, Woburn, MA, USA), following the manufacturer’s instructions. DNA was quantified using a NanoDrop ND1000 spectrophotometer (NanoDrop Technologies, Wilmington, DE, USA) and stored at −20 °C until use.

### DNA methylation analysis

We investigated the methylation level of *H19/IGF2*:IG-DMR and *KCNQ1OT1*:TSS-DMR (four CpG sites each) at the imprinted locus on chromosome 11p15.5, according to previous reports [28, 29]. Sodium bisulfite conversion of DNA samples (500 ng) was performed using the EZ DNA Methylation-Gold Kit (Zymo Research Corporation, Orange, CA, USA). PCRs were carried out using 40 ng of bisulfite-converted DNA and 200 nM forward and reverse primers, one of which was biotinylated [29]. Quantitative DNA methylation analyses were performed using a Pyro Mark ID instrument (Biotage AB, Uppsala, Sweden) in the PSQ HS 96 System (Biotage), with a PyroGold SQA reagent kit (Biotage). Hypermethylated, hypomethylated, and normal DNA samples were included in each experiment as controls. Raw data were analyzed using Q-CpG software v1.0.9 (Biotage), which calculates the ratio of converted to unconverted cytosines at each CpG, to determine the percentage of methylation. Results are presented as mean values from the analyzed CpG sites ± standard deviation (SD). Reported methylation levels represent the mean of at least two independent experiments. The statistical significance of differences in methylation among samples was determined using the Student’s t-test. We considered as “normal” *H19/IGF2*:IG-DMR and *KCNQ1OT1*: TSS-DMR methylation ranges previously reported by our group [28, 29].

### *CDKN1C* and *KCNQ1OT1*:TSS-DMR sequencing

For *CDKN1C* mutation analysis, primers were designed to amplify the two coding exons, the intron between them, and the 5′ and 3′ UTRs. PCR conditions and primers sequences have been previously described [30].

Sequencing of *KCNQ1OT1*:TSS-DMR in case 19 was performed with primers (Forward, 5′-CACGCTGTCCATAAGGTGCA-3′; Reverse, 5′-TTCTCCCAGACT Control Region 2, between the genomic positions chr11:2,699,231–2,699,440. Fragments were amplified at an annealing temperature of 55 °C, using 100 ng of template genomic DNA in 1× One Taq GC reaction buffer, 0.2 μM each primer, 0.2 mM each dNTP, and 1.25 U of One Taq DNA Polymerase (New England BioLabs, Ipswich, MA, USA). Taq High GC Enhancer One was added to the PCR to a final concentration of 20%. PCR products were analyzed by 2% agarose gel electrophoresis and purified using an UltraClean PCR Clean-Up Kit (MO BIO laboratories, Carlsbad, CA, USA), according to the manufacturer’s instructions.

Cycle sequencing reactions of both *CDKN1C* and *KCNQ1OT1*:TSS-DMR were carried out using the BigDye Terminator v3.1 Cycle Sequencing Kit (Applied Biosystems, Foster City, CA, USA) following the manufacturer’s protocol and analyzed on a 3130xl Genetic Analyzer automated 16 capillary sequencer (Applied Biosciences, Foster City, CA, USA), after purification with a ZR DNA Sequencing Clean-up Kit (Zymo Research, Irvine, CA, USA). Primers used for sequencing reactions were the same as those used for PCR. Sequences were compared to reference genomic sequence using DNA Dynamo software (Blue Tractor Software), and identified sequence variants were named according to Human Genome Variation Society recommendations (http://www.hgvs.org/mutnomen/). Nucleotide changes were numbered according to Ensembl reference sequence ENST00000414822 for *CDKN1C* and NCBI reference sequence NR_002728.3 for *KCNQ1OT1*:TSS-DMR. Functional in silico prediction of *CDKN1C* VUS was carried out using Mutation taster (www.mutationtaster.org) and PROVEAN (Protein Variation Effect Analyzer) databases.

### Array CGH

Array CGH was performed using the Agilent Technologies Platform (Santa Clara, CA, USA) with a SurePrint G3 Human CGH Microarray harboring 180,000 oligonucleotide probes.

Labeling, purification, and hybridization of DNA samples were carried out according to the manufacturer’s protocol (Agilent Oligonucleotide Array-Based CGH for Genomic DNA Analysis, version 7.3). Slides were scanned using a DNA Microarray Scanner (Agilent Technologies), and TIFF images were obtained from Agilent Scan Control software. Raw data were generated using Agilent Feature extraction and analyzed by Agilent Cytogenomics 3.0. Copy number variation analysis was performed using the ADAM2 algorithm. The aberration filter was set to detect a minimum number of three consecutive probes/region, and the minimum absolute average Log Ratio (MAALR) was ±0.25.

## Results

### Analysis of methylation of chromosome 11p15.5

**Fig. 2:**
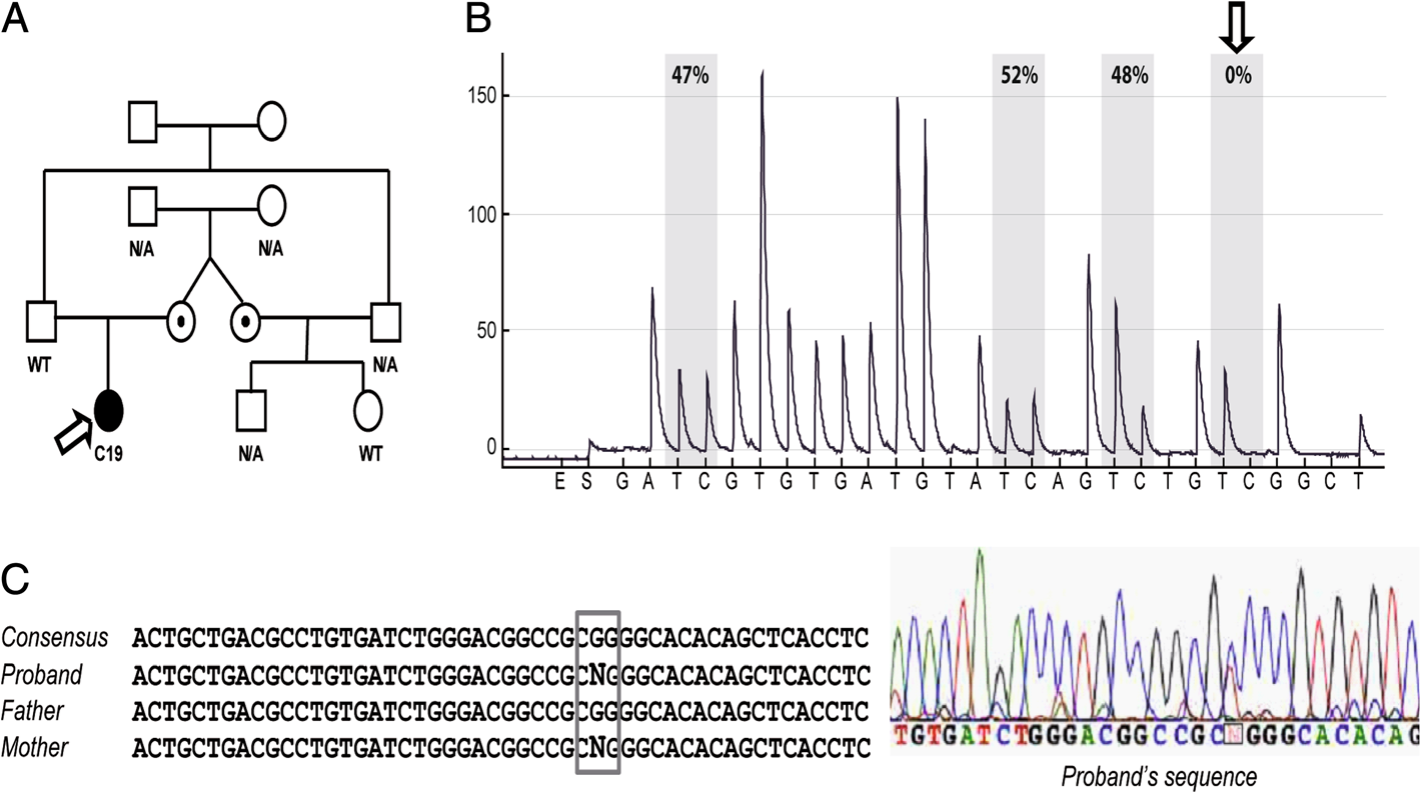
*CDKN1C* sequence variation in C19 case. In **a**, the pedigree of C19 is depicted and the proband is indicated by an arrow. As shown, the *CDKN1C* sequence variation (rs547284149) was inherited from the mother, was also present in the C19 maternal aunt but absent in her daughter who did not show omphalocele. As displayed in. **b**, this sequence variation determined a non-homogeneous methylation pattern, with a decrease in methylation at the fourth CpG site analyzed by pyrosequencing. The sequence variation and its maternal origin were confirmed by sanger sequencing of *KCNQ1OT1*:TSS-DMR: we found a heterozygous non-coding variant G > A at nucleotide 687 (NR_002728.3) (**c**)

In all samples, the methylation percentage at *H19/IGF2*:IG-DMR was within the normal range (41–51%; Table 2). At *KCNQ1OT1*:TSS-DMR, we found that 19 of 21 cases exhibited normal methylation (range: 39–50%), whereas case 2 had slight hypomethylation (38%) and case 19 (Fig. 2a) displayed a non-homogeneous methylation pattern at the fourth CpG site of the analyzed region (Fig. 2b). In the latter case we hypothesized the presence of a nucleotide variant generating an arrest in the pyrosequencing reaction of the altered strand. To explore this possibility, we performed *KCNQ1OT1*:TSS-DMR sequencing and found a heterozygous non-coding variant G > A at nucleotide 687 (NR_002728.3) (Fig. 2c). This nucleotide change affects the guanine of the fourth CpG site investigated by pyrosequencing, abolishing it and altering the global *KCNQ1OT1*:TSS-DMR methylation pattern. On the basis of the pyrosequencing methylation pattern, we hypothesized that the modified allele was of maternal origin. The pattern of inheritance was indeed confirmed in parental DNA samples by Sanger sequencing (Fig. 2a, c). The variant was also found in the healthy maternal aunt and not in her healthy daughter (Fig. 2a); thus the clinical significance of this alteration remains unknown. In addition, the same nucleotide change was identified by the 1000 Genomes project, and is described in dbSNP (ID rs547284149) with a minor allele frequency of 0.05%. Using a cut-off value of 1%, we can, therefore, define this nucleotide change as a rare variant.

**Table 2:** Methylation and sequencing analysis of 11p15.5 locus. Methylation percentage at the two 11p15.5 Differentially Methylated Regions, *CDKN1C* variants and their allelic status are reported. As already mentioned in Material and Method section, the methylation value represents the mean of the methylation percentage at each of the four analyzed CpG sites. In addition, reported methylation levels are the mean of at least two independent experiments

Case Nr	Methylation analysis		*CDKN1C* sequencing	
	*H19/IGF2:* IG-DMR (%)	*KCNQ1OT1*: TSS-DMR (%)	Genomic Variants	Alleles
1	49.3	48.0	c.555 T>C (p.A185A)	heterozygous
2	48.5	**38.6** ^**a**^	c.555 T>C (p.A185A); c.600 A > G (p.P200P)	heterozygous
3	51.0	47.0	c.511_522delGCTCCGGTCGC (p.A171_A174del)	homozygous
4	48.1	46.3	wildtype	
5	42.0	47.0	c.555 T>C (p.A185A)	heterozygous
6	47.5	47.0	c.511_522delGCTCCGGTCGC (p.A171_A174del)	heterozygous
7	47.0	49.4	wildtype	
8	47.0	45.3	wildtype	
9	49.6	48.0	wildtype	
10	49.3	47.1	wildtype	
11	49.1	47.9	c.511_522delGCTCCGGTCGC (p.A171_A174del)	heterozygous
12	44.3	45.3	wildtype	
13	50.0	50.0	wildtype	
14	44.0	45.0	c.511_522delGCTCCGGTCGC (p.A171_A174del)	homozygous
15	50.0	50.0	c.-85 G>A, c.^a^5 + 24_^a^5 + 25insG	heterozygous
16	48.0	48.0	c.555 T>C (p.A185A); c.624_629delGGCCCC (p.A209_P210del)	homozygous
17	49.0	47.0	c.555 T>C (p.A185A)	homozygous
18	45.0	49.0	c.511_522delGCTCCGGTCGC (p.A171_A174del)	heterozygous
19	41.0	dysomogeneous pattern	c.^a^5 + 24_^a^5 + 25insG	heterozygous
20	47.0	47.0	c.555 T>C (p.A185A), c.624_629delGGCCCC (p.A209_P210del)	heterozygous
21	48.0	49.0	c.511_522delGCTCCGGTCGC (p.A171_A174del)	heterozygous

^a^slightly below the normal range

### *CDKN1C* mutation analysis

Among the 21 investigated subjects, seven did not show any sequence alteration in *CDKN1C*, while 14 presented with polymorphic variants. In two of these latter cases (cases 16 and 20), an additional variant, c.624-629delGGCCCC (p.A209_P210del), was detected (Fig. 1b). This variant is located in exon 1 of *CDKN1C*, in the region encoding the PAPA domain of the protein (Table 2). Patient 16 was homozygous for the deletion (the parents were first degree cousins). Case 20 inherited the deletion from the healthy mother and the same variant was also present in the twin who died in utero and did not showed omphalocele. The deletion is reported in the dbSNP database (rs760463569) without frequency information. We sequenced 100 *CDKN1C* alleles from 50 PBL samples from healthy individuals, and the variant was not identified (data not shown). To assess the consequence of the p.A209_P210del on p57 protein function, we took advantage of the in silico predictor sites MutationTaster and Provean, and we investigated the evolutionary conservation of the entire PAPA domain by a Pairwise Sequence Alignment. Mutation Taster (www.mutationtaster.org) analysis predicted that the variant should be classified as “polymorphic” with a high probability (0.99); Provean (www.provean.jvci.org) defined the effect of the deletion as “benign” with a score of 0.489. Moreover, pairwise alignment between the nucleotides encoding the human PAPA domain and the corresponding sequence of mouse *CDKN1C* cDNA revealed a percentage of identity lower than 50% (alignment score: 315).

### Array CGH

No evidences of copy number variants with pathogenic significance were obtained (Additional file 2 A and B). The 1p31.3 duplication identified by Radhakrishna et al. [18] was not present in our cases.

## Discussion

We report the analyses of the 11p15.5 locus and the array CGH results from 21 cases with isolated omphalocele diagnosed during pregnancy or at birth. In two patients additional mild signs were present, however other specific syndromes were not suspected. Among the 19 cases with isolated omphalocele confirmed by follow-up, we observed a VUS (rs760463569) in *CDKN1C* in two cases (cases 16 and 20) and a rare variant of *KCNQ1OT1*:TSS-DMR in one case (case 19). *CDKN1C* sequencing analysis led to the identification of the same deletion c.624-629delGGCCCC in two unrelated cases, mapping inside the highly polymorphic hexanucleotide tandem-repeats of exon 1, encoding the PAPA domain of the protein. This variant is annotated in the dbSNP database (rs760463569), however its allele frequency, its clinical significance, and the segregation in the dbSNP population are not reported. Moreover, the deletion was not observed in 100 *CDKN1C* alleles derived from the 50 genotyped healthy controls, therefore it can be classified as a rare variant with unknown clinical significance (VUS). Further studies would be necessary to clarify the functional impact of this variant on the pathogenesis of the omphalocele, although we know that the PAPA domain represents a hotspot for *CDKN1C* ins/del and an extremely unstable genomic region due to the presence of repeated stretches of hexanucleotides. Moreover, the same hexanucleotide lost in the c.624-629del has been found as an insertion in healthy controls. Truncating mutations mapping at the nucleotides encoding the PAPA motif have already been described [31]. In these cases, the huge effect on gene function is likely due to the introduction of a premature STOP codon inside *CDKN1C* gene sequence.

So far, *in frame* ins/del of tandem repeated sequences in the polymorphic region of PAPA domain have never been associated with altered phenotypes. Thus, at the moment we cannot draw a conclusion about the role of the c.624-629del, even though the in silico predictions and the arguments above suggest that it represent a neutral polymorphism.

We also found a rare heterozygous maternally inherited non-coding variant G > A at the *KCNQ1OT1*:TSS-DMR locus. Interestingly, this variant alters the methylation pattern of this DMR, resulting in its partial demethylation. This nucleotide alteration was also present in a healthy maternal aunt, but not in her healthy daughter, a pattern of transmission consistent with a possible influence on the phenotype.

As mentioned above, in BWS the presence of omphalocele is mainly related to *KCNQ1OT1*:TSS-DMR hypomethylation or loss-of-function mutations in the maternally derived allele of *CDKN1C*. Thus, the presence of a VUS in 2 of 19 individuals (10.5%) and of a rare variant in one case with the same rare phenotype might not be due to a casual association, even though data supporting their causative role are not currently available. In both cases, the variant allele was maternally inherited, according to the segregation pattern associated with the pathological phenotype, but the familial segregation of the variants could not be further investigated.

On the other hand, the presence of the G>A change in the stillborn twin without omphalocele may indicate that it is not clinically significant or that its penetrance is incomplete.

## Conclusions

We can surmise that alterations of methylation levels at *H19/IGF2*:IG-DMR and *KCNQ1OT1*:TSS-DMR are not primarily associated to omphalocele, in our cohort; conversely, sequence variants at *KCNQ1OT1*:TSS-DMR and *CDKN1C* could represent susceptibility factors for the onset of isolated omphalocele. This hypothesis needs to be validated in a wider number of patients with the same clinical manifestation.

## Additional files

Additional file 1: Clinical data of pregnancies and exposition to known environmental risk factors. (XLS 23 kb)

Additional file 2: A and B: Array CGH results indicating copy number variants and their pathogenic significance. (ZIP 30 kb)

## Abbreviations

ALG6: Alpha-1,3-Glucosyltransferase
BWS: Beckwith-Wiedemann Syndrome
CDKN1C: Cyclin Dependent Kinase Inhibitor 1C
DLEU2L: Deleted In Lymphocytic Leukemia 2-Like
DMR: Differentially Methylated Region
FFPE: Formalin-Fixed Paraffin-Embedded
FOXD3: Forkhead Box D3
H19: Imprinted Maternally Expressed Transcript (Non-Protein Coding)
IG: InterGenic
IGF2: Insulin Like Growth Factor 2
IRCCS: Istituto di Ricerca e Cura a Carattere Scientifico
ITGB3BP: Integrin Subunit Beta 3 Binding Protein
KCNQ1: Potassium Voltage-Gated Channel Subfamily Q Member 1
KCNQ1OT1: KCNQ1 Opposite Strand/Antisense Transcript 1
KIAA1799: EF-Hand Calcium Binding Domain 7
MAALR: Minimum Absolute Average Log Ratio
MAPK: Mitogen-Activated Protein Kinase
OEIS: Omphalocele, Exstrophy, Imperforate anus, Spinal defects
p21^Cip1^: cyclin-dependent kinase inhibitor 1A
p27^Kip1^: Cyclin-dependent kinase inhibitor 1B
PGM1: Phosphoglucomutase 1
PHLDA: Pleckstrin Homology Like Domain Family A Member 2
ROR1: Receptor Tyrosine Kinase Like Orphan Receptor 1
SD: Standard Deviation
SLC22A18: Solute Carrier Family 22 Member 18
TSS: Transcription Start Site
UPD: Uniparental disomy
US: Ultrasound Screening
VUS: Variants of Unknown Significance
WOG: Weeks Of Gestation
